# Prune Belly Syndrome

**DOI:** 10.1155/2011/121736

**Published:** 2011-10-19

**Authors:** Koyye Ravindranath Tagore, Asok Kumar S. Ramineni, A. R. Vijaya Lakshmi, Bhavani N.

**Affiliations:** ^1^Department of Pathology, MNR Medical College, Sangareddy 502294, Andhra Pradesh, India; ^2^Department of Gynaecology & Obstetrics, MNR Medical College, Sangareddy 502294, Andhra Pradesh, India

## Abstract

Prune belly syndrome is a rare congenital disorder of the urinary system, characterized by a triad of abnormalities. The aetiology is not known. Many infants are either stillborn or die within the first few weeks of life from severe lung or kidney problems, or a combination of congenital anomalies.

## 1. Introduction

Prune belly syndrome is a congenital abnormality of unknown aetiology with characteristic features: deficient development of abdominal muscles that causes the skin of the abdomen to wrinkle like a prune, cryptorchidism, abnormalities of the urinary tract. It is associated with other congenital anomalies and most commonly clinically presented with stillborn. The case is reported for its rare congenital abnormality.

## 2. Case Report

A 22-Year-old female Gravida II, Para I Live I with 6 months of pregnancy came to hospital with decreased foetal movements for 2 days. There was no history of bleeding or draining per vagina, and no history of hypertension, diabetes, tuberculosis, bronchial asthma, and epilepsy. Also there was no history of consanguinity. Her first pregnancy was full term, normal delivery. 

 Per abdominal examination showed 34 wks of gestation with breech presentation. Foetal heart sounds present. Ultrsonography were revealed a single viable foetus with breech presentation. Foetal head deformed. There was a well-circumscribed cystic mass in the foetal abdomen measuring 15.4 × 15.4 cm. Other investigations were within normal limits.

Patient delivered a single dead foetus weighing 2.4 kg by breech presentation after aspiration of 700 mL of fluid from foetal abdomen on induction by prostaglandins. Placenta expelled out in toto. Dead foetus was subjected to pathological examination.

## 3. Autopsy Findings

### 3.1. Gross Examination

**Figure 1:**
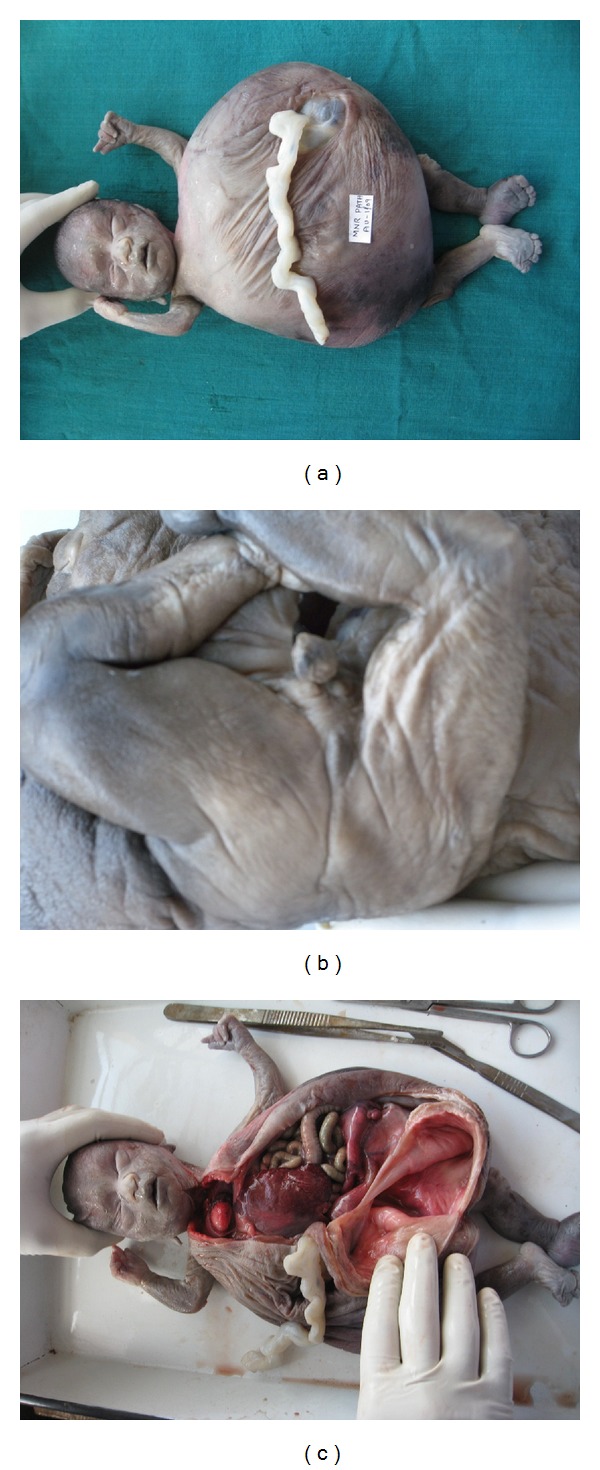
Gross findings. (a) Characteristic Potter's facies, cystic dilatation of abdomen with wrinkles and defective insertion of umbilical cord. (b) Absence of anal opening, rudimentary scrotal sac and imperforate penis. (c) Cut opened cystically dilated bladder.

Foetus of 2.4 kg weight showed potters facies consisting of ocular hypertelorism, low-set ears, receding chin, and flattening of the nose. There were absence of nipples, cystic dilatation of abdomen with deficient development of abdominal muscles, and defective insertion of umbilical cord in the anterior abdomen (Figure 1(a)). The lower extremities show club feet. Scrotal skin shows little rouge. No testes in sac. The penile urethra was not clearly made out. The anal orifice was absent (Figure 1(b)). On the back of baby was observed mild scoliosis.

On Opening the abdomen bladder measured 12 cm in diameter and was filled with straw-colored fluid. On cut section, the bladder cavity is abnormally dilated with variable thickness (Figure 1(c)). Both inner and outer surfaces of the bladder wall were smooth. No tumor was identified. The urethral orifice in the bladder is absent but both the ureteral orifices were patent. Adrenals were normal. Both kidneys were normal in anatomical location but slightly increased in size and on cut section mild dilated renal pelvicalyceal system. Both the ureters were dilated. Testes were identified in the intraabdominal position on the superior surface of the bladder on either side and each measuring 0.8 cm with short spermatic cord of 3 cm length. GIT were normal except mesentery was absent. Rectum was dilated and filled with meconiums. There was no anal orifice.

 The lungs are grossly normal number of lobes. Heart is normal and no cardiac anomalies. Were found skull and thoracic organs were normal.

### 3.2. Microscopic Examination

**Figure 2:**
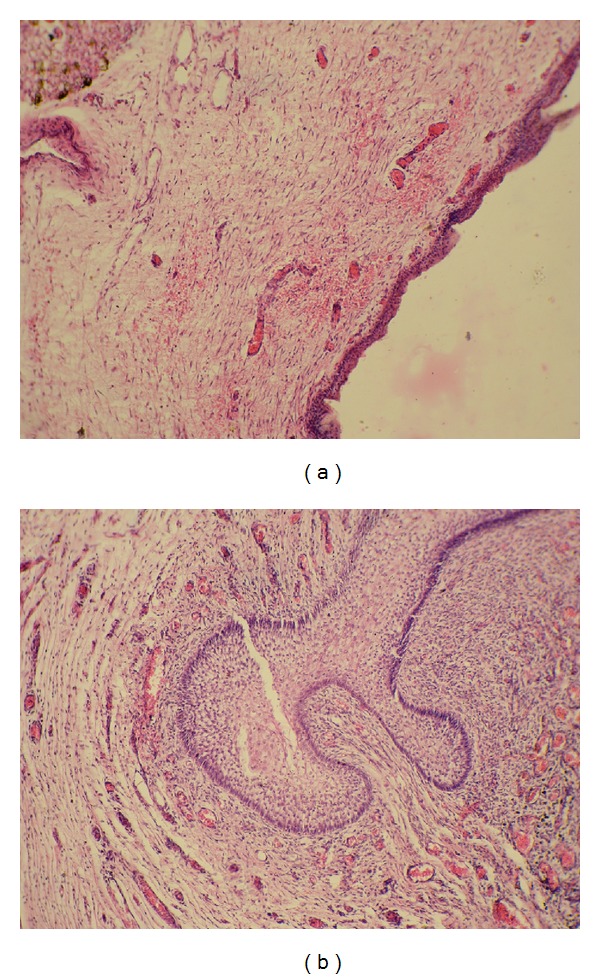
Microscopic findings. (a) Histologic section from the anterior abdominal wall showing normal skin and absence of skeletal muscle. (b) Transverse sections from the penis showing noncanalised urethra with islands of transitional epithelium and squamous metaplasia.

Sections from the anterior abdominal wall show normal skin and absence of skeletal muscle (Figure 2(a)). Both the kidneys were dysplastic with primitive tubules, fibrous tissue, and epithelial-lined cortical cysts. The bladder wall is completely replaced by fibrous tissue with hyalinization. Sections from penis show noncanalised urethra with islands of transitional epithelium and squamous metaplasia (Figure 2(b)). Sections from the remaining organs are normal.

## 4. Discussion

Prune belly syndrome is a rare congenital disorder affecting about 1 in 30,000 births [1], about 96% of those affected are male. The aetiology is not known, however some of the studies reveal the possibility of genetic inheritance [2]. In recent literature a baby was born with prune belly syndrome associated with an apparently de novo 1.3 megabase interstitial 17q12 microdeletion that includes the hepatocyte nuclear factor-1-beta gene at 17q12, and the authors suggested that haploinsufficiency of hepatocyte nuclear factor-1-beta may be causally related to the production of the prune belly syndrome phenotype through a mechanism of prostatic and ureteral hypoplasia that results in severe obstructive uropathy with urinary tract and abdominal distension [3]. Along with the classical triad of urinary tract anomalies, deficient abdominal musculature, and bilateral cryptorchidism, the prune belly syndrome is associated with a broad spectrum of defects including musculoskeletal, cardiovascular, pulmonary, and genital malformations have been documented [4]. When the urinary tract maldevelopment is associated with severe obstructive uropathy, this syndrome can lead to oligohydramnios and pulmonary hypoplasia. Our present case exhibits all classical triad of prune belly syndrome with other congenital anomalies including hypoplastic lungs, club feet, potters facies, absence of penile urethra, anal orifice, mesentery, and nipples. In the literature the incidence of clubfoot is 45%, pulmonary hypoplasia 27%, Potter facies 27%, imperforate anus 27%, and arthrogryposis (18%) [4]. In about 75% there are malformations of the cardiopulmonary, gastrointestinal, and orthopaedic systems [5]. 

The pathogenesis of prune belly syndrome is not clearly known. The mesodermal defect theory suggests that a defect exists in the mesoderm of the anterior abdominal wall and urinary tract. Between 6 and 10 weeks of gestation, aberrant development of the derivatives of the first lumbar myotome leads to a patchy muscular deficiency or hypoplasia of the abdominal wall as well as to urinary tract abnormalities [6]. An alternate theory, the urethral obstruction malformation complex, proposes that pressure atrophy of the abdominal wall muscles occurs when urethral obstruction leads to massive distension of the bladder and ureters. Bladder distension would also interfere with descent of the testes and thus be responsible for the bilateral cryptorchidism. This mechanism is responsible for the urinary tract dilatation and distension [7]. The higher incidence of this syndrome in males has been explained on the basis of the more complex morphogenesis of the male urethra, possibly resulting in obstructive anomalies at several levels. Prune belly syndrome is rare in females, with fewer than 30 cases reported in the literature [8]. Ultrasound, plain X-ray, and intravenous pyelogram are more useful investigations to diagnose the condition. Complications depend on the associated abnormalities; the most common is chronic renal failure that occurs in 25–30% of cases.

Although many ethical questions are raised when innovative fetal therapy is discussed, the insults that result from urinary tract obstruction often lead to stillbirth or neonatal death. Many infants are either stillborn or die within the first few weeks of life from severe lung or kidney problems, or a combination of congenital anomalies. There are cases of prune belly syndrome who survived into adult life after abdominal reconstruction and urinary tract repair [9]. There is no known prevention but the routine use of screening for foetal anomalies. If an antenatal diagnosis of urinary obstruction is made it may be possible to perform intrauterine surgery to prevent the development of prune belly syndrome [10]. The results seem promising.
